# Dietary methionine deficiency stunts growth and increases fat deposition via suppression of fatty acids transportation and hepatic catabolism in Pekin ducks

**DOI:** 10.1186/s40104-022-00709-z

**Published:** 2022-05-18

**Authors:** Yongbao Wu, Jing Tang, Zhiguo Wen, Bo Zhang, Junting Cao, Lulu Zhao, Zhanbao Guo, Ming Xie, Zhengkui Zhou, Shuisheng Hou

**Affiliations:** 1 State Key Laboratory of Animal Nutrition, Key Laboratory of Animal (Poultry) Genetics Breeding and Reproduction, Ministry of Agriculture and Rural Affairs, Institute of Animal Sciences, Chinese Academy of Agricultural Sciences, Beijing, 100193 China; 2 Key Laboratory of Feed Biotechnology of Ministry of Agriculture and Rural Affairs, Institute of Feed Research, Chinese Academy of Agricultural Sciences, Beijing, 100081 China

**Keywords:** Fat deposition, Hepatic catabolism, Methionine deficiency, Pekin duck, Proteomics

## Abstract

**Background:**

Although methionine (Met), the first-limiting dietary amino acid, has crucial roles in growth and regulation of lipid metabolism in ducks, mechanisms underlying are not well understood. Therefore, the objective was to use dietary Met deficiency to investigate the involvement of Met in lipid metabolism and fat accumulation of Pekin ducks.

**Methods:**

A total of 150 male Pekin ducks (15-d-old, 558.5 ± 4.4 g) were allocated into 5 groups (6 replicates with 5 birds each) and fed corn and soybean meal-based diets containing 0.28%, 0.35%, 0.43%, 0.50%, and 0.58% Met, respectively, for 4 weeks. Met-deficient (Met-D, 0.28% Met) and Met-adequate (Met-A, 0.43% Met) groups were selected for subsequent molecular studies. Serum, liver, and abdominal fat samples were collected to assess the genes and proteins involved in lipid metabolism of Pekin ducks and hepatocytes were cultured in vivo for verification.

**Results:**

Dietary Met deficiency caused growth depression and excess fat deposition that were ameliorated by feeding diets with adequate Met. Serum triglyceride and non-esterified fatty acid concentrations increased (*P* < 0.05), whereas serum concentrations of total cholesterol, low density lipoprotein cholesterol, total protein, and albumin decreased (*P* < 0.05) in Met-D ducks compared to those in Met-A ducks. Based on hepatic proteomics analyses, dietary Met deficiency suppressed expression of key proteins related to fatty acid transport, fatty acid oxidation, tricarboxylic acid cycle, glycolysis/gluconeogenesis, ketogenesis, and electron transport chain; selected key proteins had similar expression patterns verified by qRT-PCR and Western blotting, which indicated these processes were likely impaired. In vitro verification with hepatocyte models confirmed albumin expression was diminished by Met deficiency. Additionally, in abdominal fat, dietary Met deficiency increased adipocyte diameter and area (*P* < 0.05), and down-regulated (*P* < 0.05) of lipolytic genes and proteins, suggesting Met deficiency may suppress lipolysis in adipocyte.

**Conclusion:**

Taken together, these data demonstrated that dietary Met deficiency in Pekin ducks resulted in stunted growth and excess fat deposition, which may be related to suppression of fatty acids transportation and hepatic catabolism.

**Supplementary Information:**

The online version contains supplementary material available at 10.1186/s40104-022-00709-z.

## Introduction

Meat duck production has been increasing annually to support demands [1]. Over past decades, genetic selection has increased feed conversion efficiency, growth rate, and meat yield. However, this has been accompanied by excessive adipose tissues, e.g., abdominal fat, that exceeds physiological needs [2], may be harmful to ducks [3], and is not desired by producers and consumers. Consequently, excessive fat accumulation in modern strains has been one of the major and urgent problems in duck production.

Methionine (Met), an essential amino acid in diets of human and animals, regulates lipid metabolism [4, 5]. Increasing metabolic problems, especially obesity and hepatosteatosis, has increased attention on effects of Met restriction on lipid metabolism [6, 7]. In rodents, dietary Met restriction increased energy cost and reduced body fat, generally decreased serum triglyceride and cholesterol concentrations, and increased serum adiponectin [4–7]. In swine, Met deficiency reduced body weight and feed efficiency [8]; however, triglyceride concentrations in subcutaneous fat were increased, accompanied by changes in gene expressions related to lipogenesis and lipolysis [9]. Although gene expressions in pigs with dietary Met deficiency were similar to those in rodents, fat accumulation was contrary, perhaps due to dietary Met deficiency reducing daily feed intake in pigs but increasing it in rodents [10]. Furthermore, we reported dietary Met deficiency in Pekin ducks increased both daily feed intake in Pekin ducks [11], inconsistent with both pigs and rodents. In addition, whereas adipose tissue contributes more to fatty acid synthesis in pigs, the liver has a major role in lipid metabolism in ducks [12–14]. Although effects of dietary Met deficiency on lipid metabolism and fat accumulation in ducks are not well characterized, we inferred that they likely differed from pigs and rodents.

Proteomics have been used to study major nutrition-associated problems in humans and animals [15] and can facilitate discovery of key proteins regulating metabolic pathways and whose synthesis, degradation, and modifications are affected by nutrients of diet [16]. Proteomics analyses established molecular mechanisms for the roles of nutrient factors in liver. Based on 2-DE proteomics, dietary folate deficiency altered expression of hepatic proteins related to oxidative and degenerative processes [17]. Isobaric tag for relative and absolute quantitation (iTRAQ) is an emerging technique [18, 19]. The iTRAQ proteome and transcriptome analyses highlighted potential mechanisms by which dietary zinc deficiency modified abundances of genes and proteins on hepatic lipid metabolism [20]. Recently, our proteomics analyses revealed the underlying mechanisms of hepatic lipid metabolic disorders induced by dietary riboflavin deficiency causing growth depression in starter Pekin ducks [21], and embryonic death in maternal Pekin ducks [22], attributed to impaired fatty acid β-oxidation and mitochondrial electron transport chain that affected hepatic energy generation in liver. Additionally, pathogenic mechanisms of duck hepatitis A virus genotypes 3 (DHAV-3) in ducks were investigated using iTRAQ proteomics, revealing that innate immunity was triggered by DHAV-3 infection in 7-d-old Pekin ducklings, up-regulating genes and proteins related to type I interferon that caused liver damage [23].

Considering differences among mammals, rodents and poultry in fat metabolism, correlational studies on molecular mechanisms of lipid metabolism and fat deposition in mammals and rodents may not be biologically relevant for ducks. To characterize mechanisms of lipid metabolism and fat accumulation induced by dietary Met deficiency in ducks, proteomics analyses, along with RT-qPCR and Western Blot verification of related genes and proteins, were done in liver or abdominal fat of Pekin ducks fed Met-D and Met-A diets. Furthermore, hepatocytes were cultured in vitro to verify proteomics data. Therefore, this study was expected to produce evidence for the role of Met in regulating lipid metabolism of Pekin ducks.

## Materials and methods

### Animals and raising conditions

All procedures involving live animals were approved by the Animal Care and Use Committee of Institute of Animal Science, Chinese Academy of Agricultural Sciences (IAS-CAAS) and were performed according to their guidelines (IAS2018-15). A total of 180 1-d-old male Pekin ducklings (from the Pekin Duck Breeding Center of Chinese Academy of Agricultural Sciences) were fed conventional duck starter diets (ME: 12.14 MJ/kg; CP: 20%; and Met: 0.48%) for 14 d. After an overnight fast, all ducks were weighed individually at 15 days of age, and 150 ducks were selected by removing those that were lightest and heaviest. Ducks were randomized into 5 groups, each with 6 replicates and 5 birds per replicate according to similar pen body weight (558.5 ± 4.4 g). These 5 groups were kept in separate plastic-floor pens (200 cm × 75 cm × 40 cm) for 4 weeks (15 to 42 days of age) and fed a basal diet, supplemented with 5 dietary Met levels.

All ducks had ad libitum access to feed and water. Water was provided by drip-nipple water supply lines, and pelleted diet by feeders in each pen. Environmental conditions were consistent among birds and similar described previously [11], including: temperature of 32 °C and relative humidity of 20% from 1 to 3 days of age; then temperature was decreased gradually by 3 °C each week until approximately 25 °C and relative humidity was increased gradually to 65%, and thereafter temperature was approximately 18 to 22 °C. Natural light and energy-saving lighting (light intensity:10~20 lx) were provided 24 h per day.

### Experimental design and diets

A single-factor completely randomized trial design was used. Ducks were fed a basal diet supplemented with 5 levels of Met (0, 0.075%, 0.15%, 0.225%, and 0.30%, respectively). The basal diets of growing Pekin ducks were comprised mainly of corn and soybean meal (Table 1). Basal diets were divided into 5 equal sublots, and supplemented with 5 levels of crystalline DL-Met (purity ≥ 99%, Evonik Industries, Germany). The concentrations of Met and other essential amino acids in basal diet were determined with an amino acid analyzer (L-800, Hitachi, Tokyo, Japan); analyzed total Met concentrations of the 5 diets were 0.28%, 0.35%, 0.43%, 0.50%, and 0.58%, respectively. Except for Met, all other nutrients in diets met or exceeded NRC (1994) [24] nutritional requirements of White Pekin ducks from 2 to 7 weeks of age.

**Table 1:** Composition and nutrient contents of the basal diet for Pekin ducks from 15 to 42 days of age (as-fed basis)

Ingredients, %	Content	Nutrient levels	Content
Corn	68.40	Metabolizable energy^2^, kcal/kg	2973
Soybean meal	25.60	Crude protein^3^, %	16.79
Soybean oil	1.80	Methionine^3^, %	0.28
Dicalcium phosphate	1.75	Methionine + cystine^3^, %	0.56
Limestone	1.25	Lysine^3^, %	0.91
Salt	0.30	Threonine^3^, %	0.65
L-lysine•HCl	0.10	Tryptophan^3^, %	0.18
Rice hull + DL-methionine	0.30	Calcium^2^, %	0.90
Premix^1^	0.50	Total phosphorus^2^, %	0.64
Total	100.00	Non-phytate phosphorus^2^, %	0.38

^1^ Supplied per kilogram of total diet: Cu (CuSO_4_·5H_2_O), 8 mg; Fe (FeSO_4_·7H_2_O), 60 mg; Zn (ZnO), 60 mg; Mn (MnSO_4_·H_2_O), 100 mg; Se (Na_2_SeO_3_), 0.3 mg; I (KI), 0.4 mg; choline chloride, 1000 mg; vitamin A (retinyl acetate), 4000 IU; vitamin D_3_ (cholcalciferol), 2000 IU; vitamin E (dl-α-tocopheryl acetate), 20 IU; vitamin K_3_ (menadione sodium bisulfate), 2 mg; thiamin (thiamin mononitrate), 2 mg; riboflavin, 10 mg; pyridoxine hydrochloride, 4 mg; cobalamin, 0.02 mg; calcium-D-pantothenate, 20 mg; nicotinic acid, 50 mg; folic acid, 1 mg; and biotin, 0.15 mg.

^2^ Calculated values

^3^ Analyzed values

### Growth performance and carcass characteristics

At the end of the experiment (42 days of age), the weight of ducks and residual diet from each pen were measured after an overnight (10~12 h) fast. The average daily weight gain (ADG), average daily feed intake (ADFI), and feed conversion ratio (FCR) were determined for ducks from 15 to 42 days of age, with ADFI and FCR corrected for mortality. Total Met intake and Met intake per weight gain (Met intake/ADG) were calculated according to the following formula [25]: Met conversion (g/kg) = Dietary Met level (g/kg) × Feed intake (g) ÷ weight gain (g). Subsequently, 2 ducks per pen were randomly selected and weighted for assessment of carcass characteristics. Those ducks were sacrificed by neck cutting, with manual removal of feathers and evisceration. Abdominal fat, and subcutaneous fat (with skin) were removed manually from carcasses and weighed. We recorded the following weights: eviscerated carcass, abdominal fat, subcutaneous fat (with skin), with weights and percentages calculated according to the Chinese standard (NY/T 823-2004) [26].

### Sample collection

At 42 d, after overnight fasting, 2 ducks from each pen (12 birds/group) were randomly selected from 2 groups: Met-deficient group (Met-D, 0.28% Met) and Met-adequate group (Met-A, 0.43% Met). Individual blood samples of selected ducks were collected via jugular venipuncture. After 2 h at room temperature, blood samples were centrifuged (3500 × *g* for 15 min at 4 °C) and serum removed and frozen at -20 °C for determination of biochemical parameters. The ducks were sacrificed by neck cutting, and samples of liver and abdominal fat (from consistent locations) were quickly collected, and frozen in liquid nitrogen and stored at -80 °C until further analysis for proteomics, Western blot, and qRT-PCR. Subsequently, liver and abdominal fat tissues (consistent locations) were fixed in 4% paraformaldehyde for histology and remaining tissues were stored at -20 °C for analysis of hepatic lipids.

### Serum biochemical parameters

Serum non-esterified fatty acid (NEFA) concentrations were measured with commercial kits (Nanjing Jiancheng Bio-engineering Institute, code no. A042, Nanjing, China) according to the manufacturer’s instructions. Other serum biochemical parameters were determined with an automatic biochemical analyzer (Hitachi 7080 Automatic Aralyzer, Tokyo, Japan), using corresponding kits (Maccura Biotechnology Co. LTD, Chengdu, China).

### Hematoxylin-eosin staining

After fixation in 4% paraformaldehyde, liver and abdominal fat tissues were dehydrated in increasing concentrations of alcohol and embedded in paraffin. Sections were cut (4-5 μm thickness), stained with Hematoxylin-Eosin (H&E), and observed under light microscopy with 100× or 400× magnification (Olympus, Tokyo, Japan).

### Hepatic lipid concentration

Hepatic total lipids were extracted by homogenizing minced liver samples in chloroform-methanol (2:1, vol/vol), as described [27]. The extracts were evaporated under a stream of nitrogen, weighed, and resuspended in 4 mL chloroform-methanol (2:1) solution containing 0.01% butylated hydroxytoluene [21]. Concentrations of triglyceride (TG) and total cholesterol (TCHO) were measured with commercial assay kits (Nanjing Jiancheng Bio-engineering Institute, code no. A111&A112, Nanjing, China). Liver samples were weighed and placed in a lyophilizer (LGL-10D, Beijing, China). After freeze-drying, samples were weighed and ground to pass through a 40-mesh sieve. Hepatic fatty acid concentrations were analyzed as described previously [28] and reported as mg/g liver.

### Hepatic proteomics

Three biological replicates (4 liver samples were combined into 1 biological replicate) from each group were used for iTRAQ assays. Hepatic protein was extracted as described [23] and protein concentrations determined (BCA assay reagent kit; Pierce, Thermo Scientific, Rockford, Illinois, USA), following manufacturer’s recommendations. A consistent amount of protein (100 μg) from each sample was digested and labelled with iTRAQ 8-plex reagents (AB Sciex Inc., Foster City, California, USA) according to the manufacturer’s instructions (Met-D1, Met-D2, Met-D3, Met-A1, Met-A2, and Met-A3 were labeled with 113, 114, 115, 116, 117, and 119, respectively). The labeled samples were combined and fractionated into 12 fractions by HPLC (Thermo DINOEX, USA) using a Durashell C18 column (5μm, 100 Å, 4.6 mm ×250 mm). Liquid chromatography-electrospray ionization-MS/MS was operated with a Triple TOF 5600 plus system (AB SCIEX, USA).

Data for the original MS/MS files identification and quantification were analyzed against the database Uniprot-taxonomy-Anas-platyrhynchos.fasta using ProteinPilot Software V4.5 (AB Sciex, USA). Only unique peptides with ≥ 95% confidence intervals were contained in the iTRAQ labelling quantification and used for further analysis. To identify differentially expressed proteins, relative protein expression values were compared between groups (Met-D vs. Met-A). Proteins were regarded as differentially expressed if the iTRAQ ratios were ≥ 1.2 or ≤ 0.83 with *P* <0.05, as analyzed with a by the paired Student’s *t*-test. Both GO annotation and KEGG pathway enrichment analysis of differentially expressed proteins were used to determine functional subcategories and metabolic pathways.

### Hepatocyte culture and treatment

Both sources of hepatic cells, including HepG2 cell line (Cell Bank of Chinese Academy of Sciences) and duck primary hepatocytes (isolated from duck embryos, as described [29]), were cultured in Dulbeccos's modified Eagle's medium (DMEM, Gibco) containing 10% fetal bovine serum (FBS, Gibco), 1% penicillin-streptomycin (Gibco), with 5% CO_2_ at 37 °C. The HepG2 cells were cultured in 6-well plates and starved for 6 h after reaching 90% confluence. Subsequently, the HepG2 cells were incubated in the Met-free basal DMEM medium (control, 0 μmol/L L-Met) and the basal medium supplemented with 25 or 200 μmol/L L-Met (Sigma) for 24 h, respectively. The duck primary hepatocytes were cultured in 6-well plates in the Met-free basal DMEM medium supplemented with 0, 25 or 200 μmol/L L-Met for 24 h, respectively, after starving for 6 h. Both kinds of hepatic cells were harvested and lysed in PIPA buffer, and frozen at -80 °C for further analysis of ALB protein expressions.

### Quantitative reverse transcription PCR analysis

Total RNA of liver, abdominal fat, and hepatocyte samples was isolated with RNAiso Plus (TaKaRa, Code no. 9109, Dalian, China), following manufacturer’s instructions. The total RNA concentration of each sample was measured by determining absorbance at 260 nm (Thermo Fisher Scientific, NanoDrop 2000, Waltham, USA) and RNA integrity was evaluated by agarose gel electrophoresis. Then, RNA samples were reverse-transcribed to complementary DNA (cDNA) according to the manufacturer’s instructions (PrimerScriptTM RT Master Mix, TaKaRa, Code no. RR036A, Dalian, China), as follows: incubation at 37 °C for 15 min, followed by 85 °C for 5 s. Quantitative Reverse Transcription (qRT) PCR analysis was performed in a fluorescence detection system (Bio-Rad CFX384TMReal-Time PCR, USA), with these cycling conditions: 30 s at 95 °C for denaturation, 5 s at 95 °C, 34 s at 60 °C (40 cycles), 15 s at 95 °C, and a final step for 1 min at 60 °C. Primers were designed using Primer Premier 6 software (Table S1), and synthesized commercially (Tsingke Biotech, Beijing, China). Beta-actin was the internal reference gene, due to its stable expression in Pekin ducks (*Anas platyrhnchos*). The PCR amplification efficiency for each primer ranged from 90% to 110%, with each sample analyzed in triplicate. Relative mRNA expression of target genes was determined using the 2^−ΔΔCt^ method [30].

### Western blotting

Total proteins from liver, abdominal fat, and hepatocytes were lysed in PIPA buffer containing 1% protease inhibitor and 1% phosphorylase inhibitors, centrifuged at 12,000 × *g* at 4 °C for 15 min, and supernatant collected. Total protein concentrations were determined using a BCA protein assay kit (Pierce, Thermo Scientific, USA). Equal quantities of total protein (25 μg per lane) were separated on 10% SDS-PAGE gels, and blotted onto polyvinylidene difluoride membrane (0.45 μm, Pall, USA). After being blocked in 5% skim milk for 2 h, the membrane was incubated with primary antibodies overnight at 4 °C. Thereafter, the membrane was incubated with the appropriate HRP-labeled secondary antibody for 1.5 h at room temperature. Immuno-reactive proteins were visualized using a chemiluminescence detection kit (Thermo Fisher Scientific, USA). An imaging system (Tanon Gis) and ImageJ software (NIH, Bethesda, MD, USA) were used to determine the blot signal and protein density. Primary antibodies against medium-chain specific acyl-CoA dehydrogenase (ACADM) (ab92461, Abcam, 1:5000), NADH dehydrogenase (ubiquinone) Fe-S protein 1 (NDUFS1) (ab169540, Abcam, 1:5000), albumin (ALB) (AP21444SU-N, OriGene,1:2500), lactate dehydrogenase (LDH) (WL03271, Wanleibio,1:2000), fatty acid-binding protein 1 (FABP1) (A5311, Abclonal, 1:2500), fatty acid synthase (FASN) (A0461, Abclonal, 1:2500), adipose triacylglyceride lipase (ATGL) (A6245, Abclonal, 1:2500), hormone-sensitive lipase (HSL) (A15686, Abclonal, 1:2500), and phosphor-hormone-sensitive lipase at Ser563 (p-HSL-S563) (AP0851, Abclonal, 1:2500) were used. All results were expressed as the abundance of each protein relative to beta-tubulin (HX1829, HuaxingBio, 1:5000), and relative expression was normalized.

### Statistical analyses

Data were analyzed by the one-way ANOVA with GLM procedure or Student’s *t*-test (SAS Version 9.4). Values were considered significant if *P* < 0.05. For growth performance and carcass traits, Duncan’s multiple comparison tests were used to compare differences among means if there were differences in dietary Met level, whereas linear or quadratic effects of dietary Met level were analyzed by orthogonal polynomials only when the main effect was significant (*P* < 0.05). A Student’s *t*-test was used to analyze serum biochemistry, qRT-PCR, and Western Blot. All data were expressed as Mean ± standard deviation.

## Results

### Growth performance and carcass characteristics

Dietary Met content of basal diet was analyzed to be 0.28% (Table 1), consistent with the calculated value. Ducks fed the basal diet had growth depression (lowest ADG and highest FCR), which were ameliorated by diets with Met supplementation (Table 2). The ADG of ducks increased in a linear manner (*P* < 0.05), whereas the FCR decreased in linear and quadratic manners (*P* < 0.05) as dietary Met content increased. Meanwhile, Met intake increased in a linear manner and the ratio of Met intake to ADG (Met intake/ADG) increased in linear and quadratic manners as dietary Met increased.

**Table 2:** Effects of dietary methionine level on growth performance of Pekin ducks from 15 to 42 days of age

Dietary Met level, %	ADG, g	ADFI, g	FCR	Met intake, g/d/bird	Met intake/ADG
0.28	86.7±2.6^b^	186.2±7.9	2.15±0.06^a^	0.52±0.02^e^	5.95±0.16^e^
0.35	89.1±3.6^a,b^	176.7±10.2	1.98±0.06^b^	0.62±0.04^d^	6.97±0.20^d^
0.43	93.4±2.6^a^	179.1±8.2	1.92±0.04^c^	0.76±0.03^c^	8.19±0.16^c^
0.50	90.8±3.0^a,b^	175.5±7.2	1.94±0.04^b,c^	0.88±0.04^b^	9.70±0.19^b^
0.58	91.8±4.6^a^	177.0±9.6	1.93±0.04^b,c^	1.02±0.06^a^	11.12±0.25^a^
*P* value					
Met	0.024	0.259	<0.001	<0.001	<0.001
Met linear response	0.014		<0.001	<0.001	<0.001
Met quadratic response	0.077		<0.001	0.489	0.001

^a–e^ Means within a column with no common superscripts differ significantly (*P* < 0.05)

Values are the means of 6 replicates of 5 ducks

*ADG*, average daily gain; *ADFI*, average daily feed intake; *FCR*, feed conversion ratio (feed: gain); *Met*, methionine

A basal diet without Met supplementation had negative effects on carcass traits of growing ducks (Table 3), although the weight and percentage of eviscerated carcasses both increased in linear and quadratic manners (*P* < 0.05) as dietary Met increased. Furthermore, abdominal fat weight and percentage, skin and subcutaneous fat percentage had linear and quadratic declines (*P* < 0.05) in response to increasing dietary Met. However, dressing percentage was not affected (*P* > 0.05) by dietary Met supplementation.

**Table 3:** Effects of dietary methionine level on carcass traits of Pekin ducks at 42 days of age.

Dietary Met level, %	Dressing percentage, %	Eviscerated carcass		Abdominal fat		Skin and subcutaneous fat	
		Weight, %	Percentage, %	Weight, %	Percentage, %	Weight, %	Percentage, %
0.28	86.8±1.0	1663±105^b^	57.9±2.2^b^	32.2±10.6^a^	1.46±0.48^a^	514.0±94.5	25.0±3.7^a^
0.35	87.0±1.6	1712±115^b^	59.1±1.6^b^	26.6±5.9^b^	1.19±0.26^b^	492.1±46.4	23.5±1.4^a,b^
0.43	86.8±0.9	1858±142^a^	60.9±1.8^a^	20.3±2.8^c^	0.86±0.11^c^	489.9±37.6	21.8±1.7^b^
0.50	87.5±1.0	1777±177^a,b^	61.8±1.6^a^	20.1±5.6^c^	0.89±0.21^c^	454.6±81.9	21.6±1.2^b^
0.58	87.2±1.0	1775±129^a,b^	60.8±1.5^a^	20.1±5.8^c^	0.85±0.21^c^	473.8±66.2	21.6±2.1^b^
*P* value							
Met	0.492	0.013	<0.001	<0.001	<0.001	0.301	0.001
Met linear response		0.026	<0.001	<0.001	<0.001		<0.001
Met quadratic response		0.032	0.007	0.019	0.011		0.071

^a–c^ Means within a column with no common superscripts differ significantly (*P* < 0.05).

Values are the means of 6 replicates of 2 ducks.

The Met requirements of growing Pekin ducks were estimated by broken-line regression models (Table S2). Dietary Met requirement of Pekin ducks from 15 to 42 days of age for optimal FCR, abdominal fat weight, abdominal fat percentage, and skin and subcutaneous fat percent were 0.374%, 0.431%, 0.427%, and 0.438%, respectively, based on the linear-broken line model, and dietary Met requirement was 0.420% for optimal FCR, based on the quadratic broken-line regression model. Therefore, dietary Met requirement of growing Pekin ducks from 15 to 42 days of age was approximately 0.43% in the present corn-soybean meal diet.

### Serum biochemical parameters

**Fig. 1:**
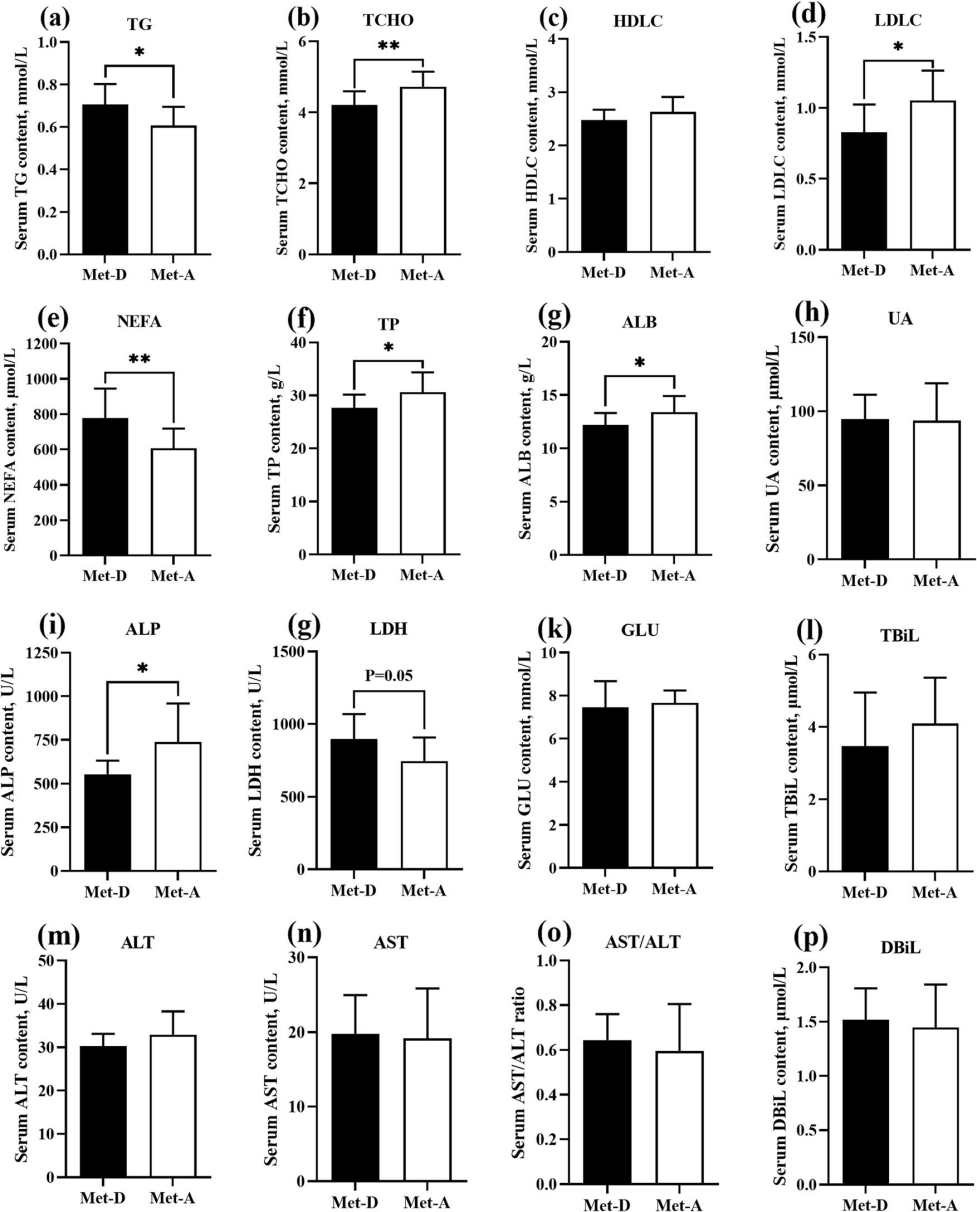
Serum biochemical parameters of Pekin ducks in Met-deficient and Met-adequate groups. Results are presented as means with plus error bars (standard deviation). Differences were assessed by Student’s *t* test (*n* = 12) and denoted as follows: * *P* < 0.05; ** *P* < 0.01. Met-D, Met-deficient; Met-A, Met-adequate; TCHO, Total cholesterol; TG, Triglyceride; HDLC, High-density lipoprotein cholesterol; LDLC, Low-density lipoprotein cholesterol; NEFA, Non-esterified fatty acid; TP, Total protein; ALB, Albumin; ALT, Alanine aminotransferase; AST, Aspartate amino transferase; TBIL, Total bilirubin; DBIL, Direct bilirubin; ALP, Alkaline phosphatase; LDH, Lactic dehydrogenase; UA, Uric acid; GLU, Glucose

Ducks fed a Met-deficient diet had greater TG and NEFA (*P* < 0.05), and lower serum TCHO and LDLC concentrations (*P* < 0.05) compared to birds in the Met-A group (Fig. 1). In addition, the TP, ALB and ALP concentrations in serum were decreased (*P* < 0.05), and LDH content was increased (*P* = 0.05) in the Met-D group.

### H&E staining and liver lipids

**Fig. 2:**
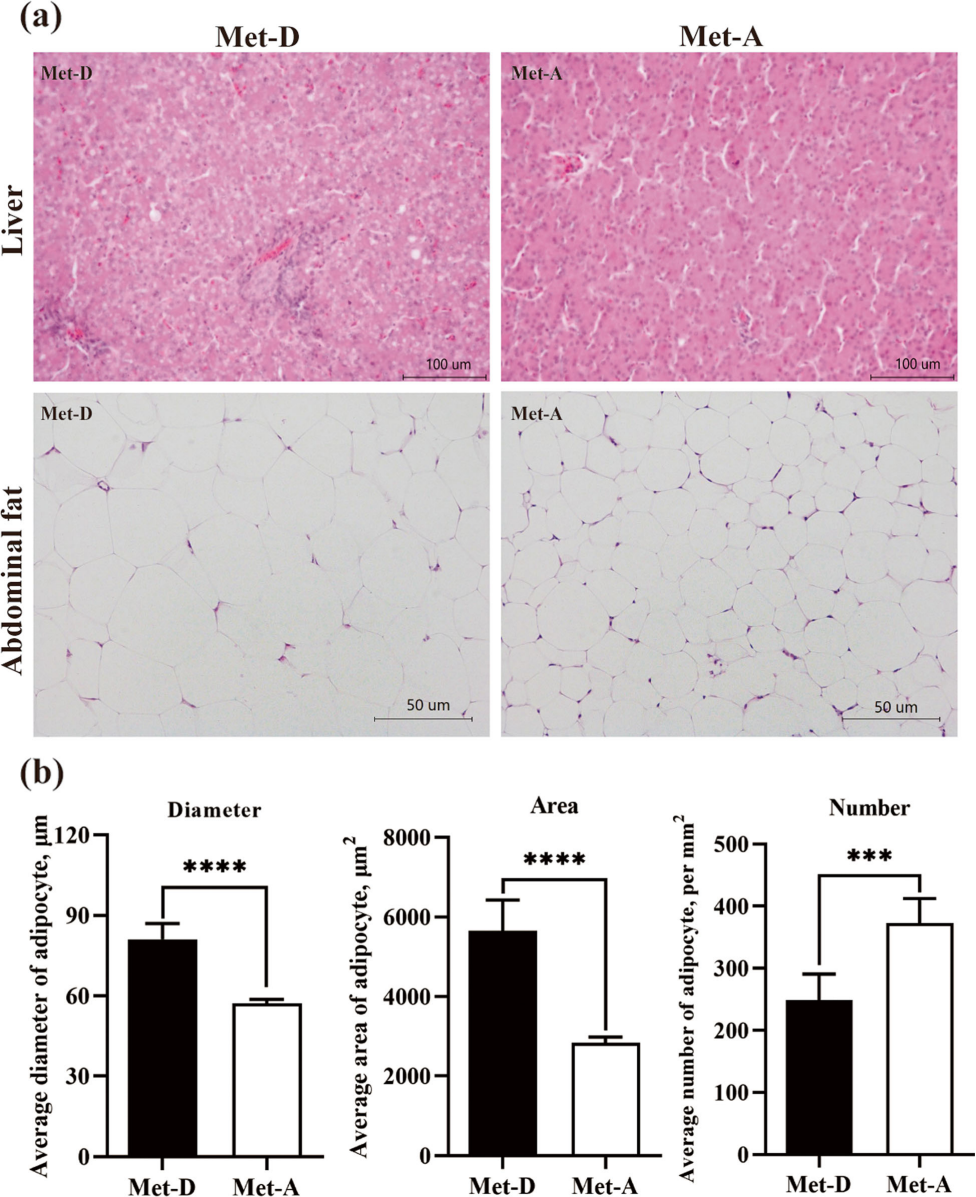
Fat deposition in Pekin ducks in Met-deficient and Met-adequate groups. **a** Hematoxylin and Eosin (H&E) staining of liver and abdominal fat. Photomicrographs are shown at 400× and 200× for liver and for abdominal fat, respectively. **b** The average number, diameter, and area of adipocytes in abdominal fat. Results are presented as means with plus error bars (standard deviation). Differences were assessed by Student’s *t* test (*n* = 6) and denoted as follows: *** *P* < 0.001, **** *P* < 0.0001. Met-D, Met-deficient; Met-A, Met-adequate

Histological characteristics of the liver and abdominal fat were determined based on H&E staining. There were increases in cell area and diameter (*P* < 0.05), and a decrease in cell count (*P* < 0.05) of abdominal adipocytes from Pekin ducks fed a Met-deficient diet (Fig. 2b). On H&E staining, the liver had small lipid droplets in Met-D groups (Fig. 2a), whereas total lipids, TG and TCHO concentrations and fatty acid compositions were not significantly different between Met-D and Met-A groups (Fig. S1).

### Hepatic proteomics

**Fig. 3:**
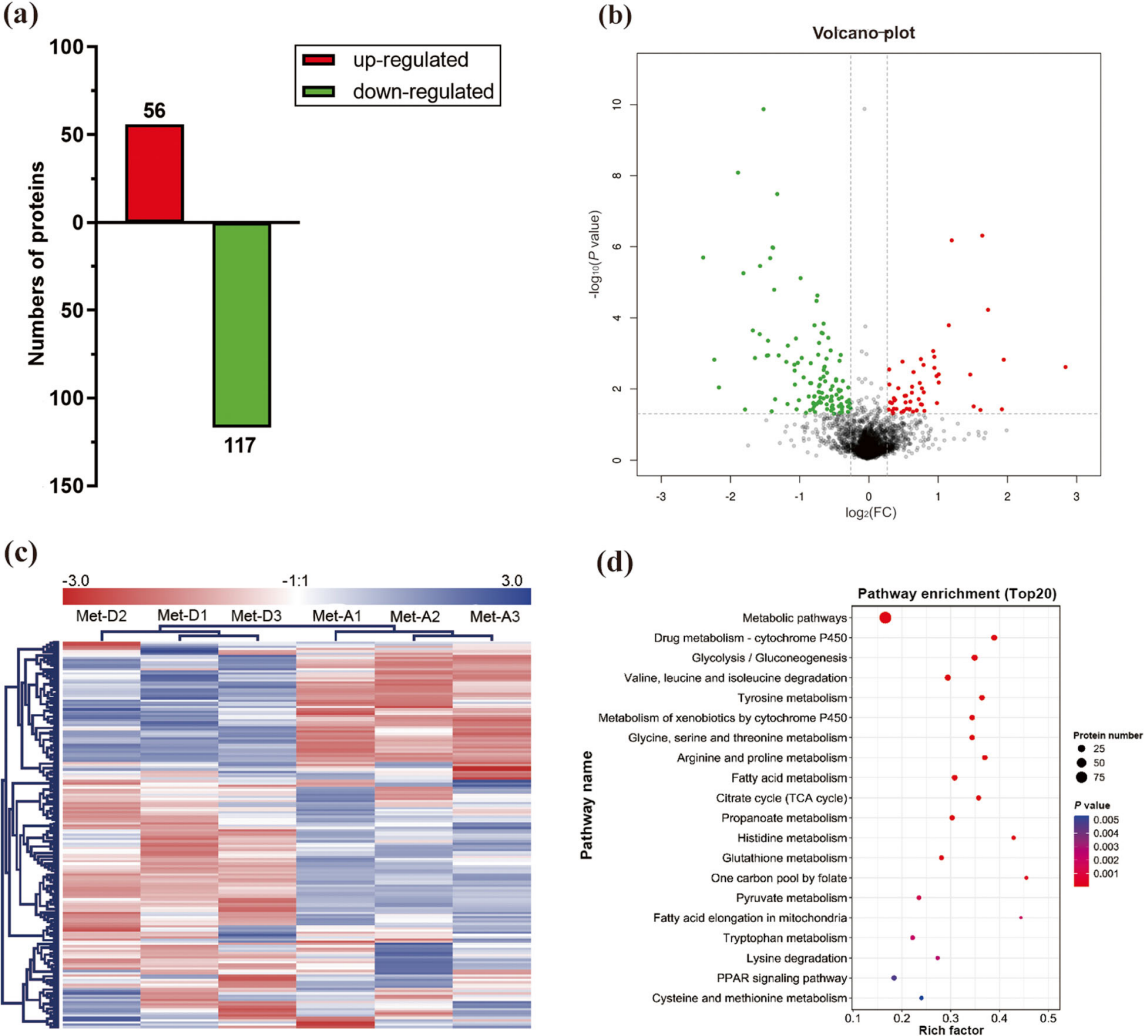
Differentially expressed proteins in the liver of Pekin ducks in Met-deficient and Met-adequate groups. The (**a**) number, (**b**) volcano plot, (**c**) heatmap, and (**d**) KEGG signal pathway of differentially expressed proteins. Met-D, Met-deficient; Met-A, Met-adequate

In the iTRAQ analysis, 2871 proteins were identified from 19,859 peptide spectral matches in liver of Met-D and Met-A groups. Among identified proteins, there were 173 differentially expressed proteins (fold-change ≥ 1.2 or ≤ 0.83, *P* < 0.05), including 56 up-regulated proteins and 117 down-regulated proteins, after comparing relative abundance of proteins in livers of Pekin ducks fed Met-D and Met-A diets (Fig. 3a-c). The complete list of all differentially expressed proteins is shown in Table S3. Based on KEGG enrichment analysis of differentially expressed proteins, 35 pathways were significantly enriched (Fig. 3d; Table S4), including those mainly focused on nutrient metabolism, namely metabolic pathways (*P* = 2.85E-22), glycolysis/gluconeogenesis (*P* = 4.36E-8), fatty acid metabolism (*P* = 4.86E-6), tricarboxylic acid cycle (TCA cycle, *P* = 6.96E-6), pyruvate metabolism (*P *= 3.64E-5), and cysteine and Met metabolism (*P* = 0.005). Furthermore, PPAR signaling pathway closely related to lipid metabolism was enriched (*P* = 5.0E-3). Since lipid metabolism and energy generation were mainly impacted by dietary Met deficiency, differentially expressed proteins related to both above-mentioned processes were classified into 6 categories that were associated with fatty acid transport, fatty acid oxidation, electron transport chain, TCA cycle, glycolysis/gluconeogenesis, and ketoplasia processes, respectively (Table 4). Furthermore, the majority of differentially expressed proteins related to those processes were down-regulated (Table 4).

**Table 4:** The main differentially expressed proteins in liver of Pekin ducks induced by dietary methionine deficiency

Accession	Gene name	Protein name	Fold change	*P* value
**Fatty acid transport**				
U3I073	*FABP1*	Fatty acid binding protein 1	3.29	5.9E-05
U3J4Z9	*ACSL5*	Acyl-CoA synthetase long chain family member 5	0.72	5.9E-03
U3I2B5	*SLC27A5/FATP5*	Solute carrier family 27 member 5	0.62	2.4E-02
U3ILY2	*ACBP*	Acyl-CoA-binding protein	0.61	1.2E-02
U3IU92	*ALB*	Albumin	0.35	1.3E-10
U3IKU2	*SCP2*	Sterol carrier protein 2	0.48	4.0E-04
**Fatty acid oxidation**				
R0LHZ1	*ANAPL_13096*	3-ketoacyl-CoA thiolase, mitochondrial (Fragment)	0.60	1.2E-02
U3J928	*ACOX1*	Acyl-coenzyme A oxidase	0.60	1.1E-03
U3I806	*HADHA*	Hydroxyacyl-CoA dehydrogenase trifunctional multienzyme complex subunit alpha	0.63	3.0E-04
U3IIH2	*HADH*	Hydroxyacyl-CoA dehydrogenase	0.58	1.7E-02
U3ITA9	*ACADM*	Acyl-CoA dehydrogenase medium chain	0.59	3.3E-05
R0JMW7	*ANAPL_02624*	Fatty aldehyde dehydrogenase (Fragment)	0.58	2.7E-02
U3IHG8	*ALDOB*	Fructose-bisphosphate aldolase	0.58	3.1E-02
**Electron transport chain**				
U3I998	*NDUFS1*	NADH:ubiquinone oxidoreductase core subunit S1	0.76	3.7E-02
U3J7F4	*ETFA*	Electron transfer flavoprotein alpha subunit	0.57	2.5E-02
R0LSV8	*ETFDH*	Electron transfer flavoprotein-ubiquinone oxidoreductase, mitochondrial (Fragment)	0.74	1.6E-03
**TCA cycle**				
U3IR48	*DLD*	Dihydrolipoyl dehydrogenase	0.41	1.1E-03
U3ICQ7	*DLAT*	Acetyltransferase component of pyruvate dehydrogenase complex	0.61	1.8E-02
R0L7Q0	*ANAPL_09620*	Fumarate hydratase (Fragment)	0.56	1.9E-03
U3J597	*IDH1*	Isocitrate dehydrogenase [NADP]	0.52	4.8E-03
U3J925	*ACLY*	ATP-citrate synthase	0.50	7.6E-06
U3IR57	*MDH1*	Malate dehydrogenase 1	0.62	2.0E-04
U3IA60	*MDH2*	Malate dehydrogenase 2	0.63	1.0E-04
**Glycolysis/Gluconeogenesis**				
U3J1L1	*GAPDH*	Glyceraldehyde-3-phosphate dehydrogenase	0.48	3.0E-03
U3ILF5	*PGK1*	Phosphoglycerate kinase	0.38	1.1E-06
U3J2H8	*FBP1*	Fructose-bisphosphatase 1	0.36	4.0E-04
U3I8D8	*TPI1*	Triosephosphate isomerase	0.32	1.3E-03
U3IE74	*LDHA*	L-lactate dehydrogenase	3.11	4.9E-07
P13743	*LDHB*	L-lactate dehydrogenase B chain	0.64	2.4E-03
**Ketoplasia**				
U3J9A9	*HMGCS2*	3-hydroxy-3-methylglutaryl coenzyme A synthase	0.31	2.0E-04

### qRT-PCR and Western blotting

**Fig. 4:**
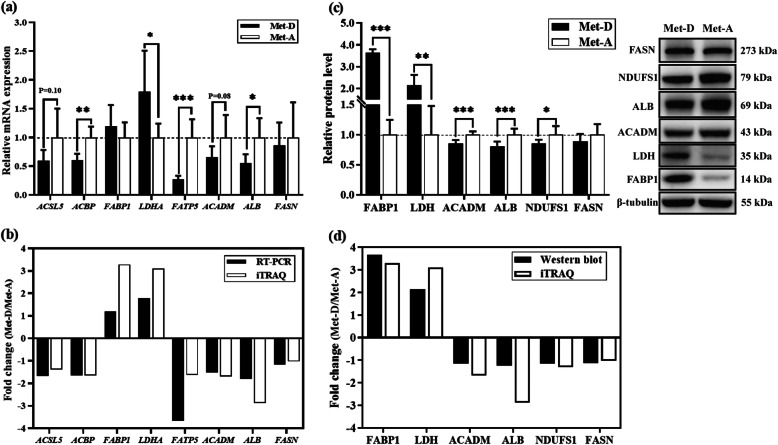
Verification of hepatic proteomic analyses in Pekin ducks by Western blot and real-time PCR. The mRNA abundances were determined by real-time PCR analysis (**a**) and protein expression determined by Western Blot (**b**) in liver; (**c, d**) fold change of selected proteins between real-time PCR, Western blot validation and proteomics analysis. Results are presented as means with plus error bars (standard deviation). Differences were assessed by Student’s *t* test (*n* = 6) and denoted as follows: * *P* < 0.05; ** *P* < 0.01; *** *P* < 0.001. Met-D, Met-deficient; Met-A, Met-adequate. FABP1 (L-FABP), Fatty acid-binding protein, liver; ACBP, Acyl-CoA-binding protein; ACSL5, Acyl coenzyme A long-chain 5 synthetase; FATP5, Fatty acid transport protein 5; LDHA, Lactate dehydrogenase A chain; FASN, Fatty acid synthase; NDUFS1, NADH:ubiquinone oxidoreductase core subunit S1; ACADM, Acyl-CoA dehydrogenase medium chain; ALB, Albumin

Both qRT-PCR and Western blot analysis of several key proteins were done to validate results of iTRAQ proteomics. The mRNA expressions of acyl-coenzyme A binding protein (*ACBP*, *P* < 0.01), fatty acid transport protein 5 (*FATP5*, *P* < 0.001), albumin (*ALB*, *P* < 0.05), long-chain acyl-CoA dehydrogenase (*ACADM*, *P* = 0.083), and long-chain acyl-CoA synthase 5 (*ACSL5*, *P* = 0.10) were diminished, whereas lactate dehydrogenase A (*LDHA*, *P* < 0.05) was enhanced in liver of Pekin ducks fed a Met-deficient diet, which suggested that the majority of key proteins had similar expression patterns at both transcriptional and protein levels (Fig. 4a,b). The hepatic protein expressions of NADH: ubiquinone oxidoreductase core subunit S1 (NDUFS1, *P* < 0.05), ACADM (*P* < 0.001) and ALB (*P* < 0.01) were diminished, whereas fatty acid binding protein1 (FABP1, *P* < 0.001) and LDH (*P* < 0.01) were enhanced in Pekin ducks fed a Met- deficient diet, consistent with the results of iTRAQ proteomics (Fig. 4c,d).

**Fig. 5:**
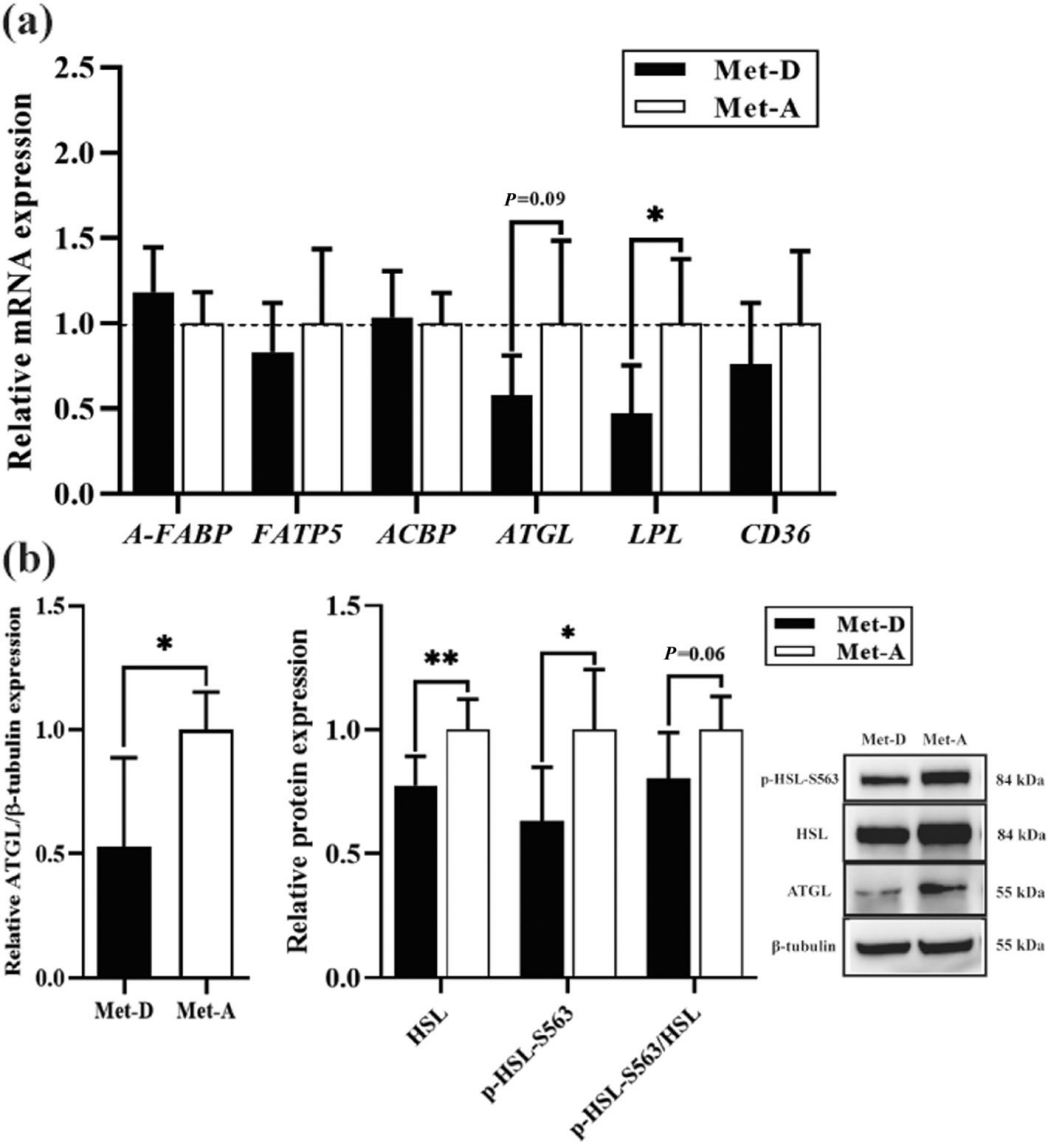
Relative mRNA and protein expression in abdominal fat of Pekin ducks in Met-deficient and Met-adequate groups. Results are presented as means with plus error bars (standard deviation). Results are presented as means with plus error bars (standard deviation). Differences were assessed by Student’s *t* test (*n* = 6) and denoted as follows: * *P* < 0.05; ** *P* < 0.01; *** *P* < 0.001. Met-D, Met-deficient; Met-A, Met-adequate; A-FABP (FABP4), Fatty acid-binding protein, adipose; ACBP, Acyl-CoA-binding protein; FATP5, Fatty acid transport protein 5; LPL, Lipoprotein lipase; ATGL, Adipose triacylglyceride lipase; FAT/CD36, Fatty acid translocase; HSL, Hormone-sensitive lipase; p-HSL-S563, Phosphor-hormone-sensitive lipase at Ser563

The qRT-PCR and Western blot analyses of genes and proteins associated with lipolysis were done in abdominal fat. Dietary Met deficiency reduced the reduction of lipoprotein lipase (*LPL*, *P* < 0.05) and adipose triglyceride lipase (*ATGL*, *P* = 0.09) mRNA expression (Fig. 5a). Similarly, protein expressions of ATGL (*P* < 0.05), hormone-sensitive lipase (HSL, *P* < 0.05), phosphorylated HSL at Ser563 (p-HSL-S563, *P* < 0.05), and the ratio of p-HSL-S563 to HSL (p-HSL-S563/HSL, *P* = 0.06) were all decreased in abdominal fat in ducks fed a Met-deficient diet (Fig. 5b).

### Hepatocyte in vitro verification

**Fig. 6:**
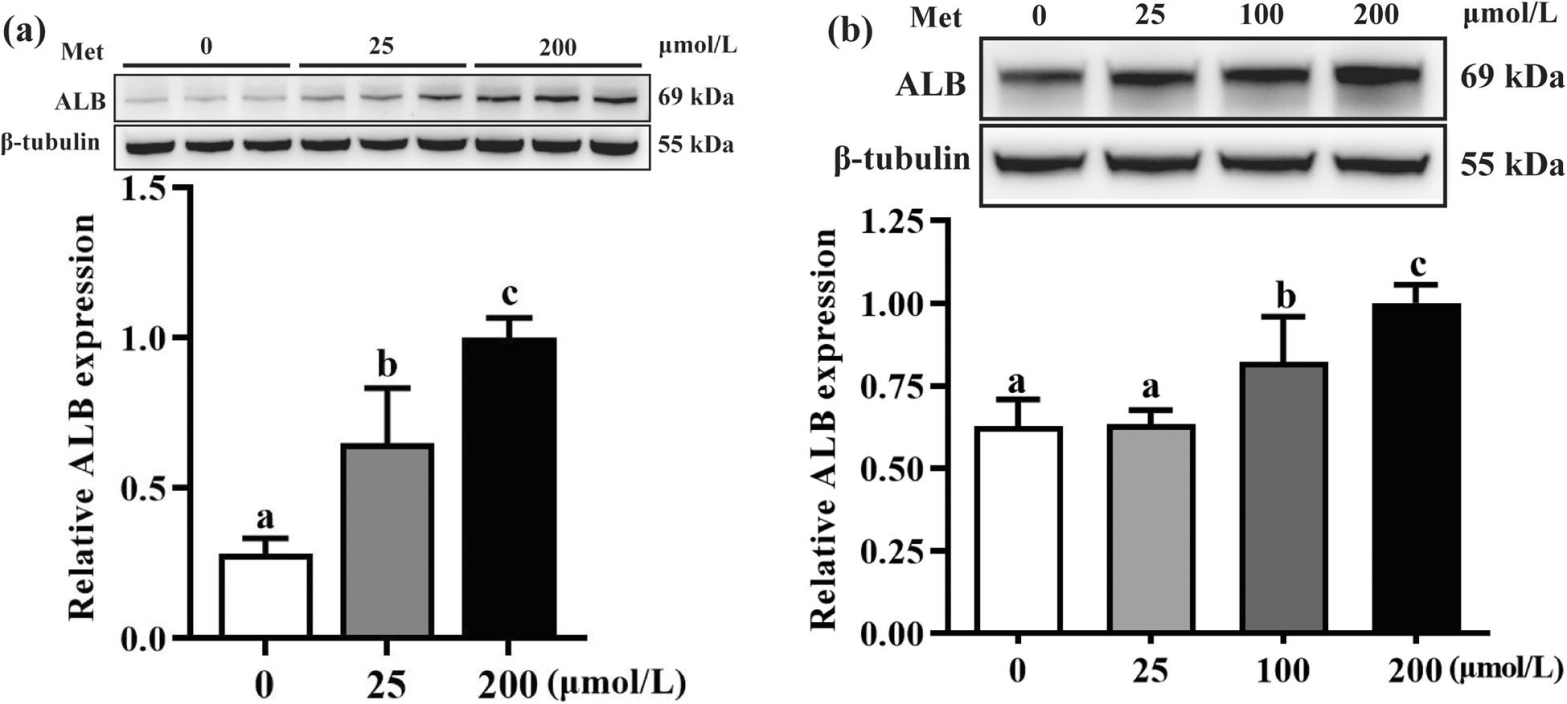
Relative albumin expression in hepatocytes with various Met concentrations in medium. **a** HepG2 (*n* = 3) and (**b**) duck primary hepatocytes (*n* = 4). Results are presented as means with plus error bars (standard deviation). Differences were assessed by one-way analysis of variance, and values with different letter superscripts mean significant difference at *P* < 0.05. ALB, albumin

Hepatocytes, including the HepG2 cell line and duck primary hepatocytes, were cultured in vitro in DMEM medium with varying different Met concentrations, and the ALB protein expression in both hepatocytes were determined to validate the proteomic analysis. The ALB protein expression of HepG2 cell in Met-deficient groups (0, 25 μmol/L) were decreased (*P* < 0.05) compared to the Met-adequate group (200 μmol/L) (Fig. 6a). Equally, compared to the control group (200 μmol/L), ALB protein expression in duck primary hepatocyte were decreased (*P* < 0.05) when lower Met supplementation (0, 25, and 100 μmol/L) were added to DMEM medium (Fig. 6b). These patterns of ALB expression in hepatocytes in vitro were in accordance with proteomic analyses in liver of Pekin ducks.

## Discussion

As Met is considered the first limiting amino acid in corn-soybean meal diets for ducks, crystalline Met sources, e,g. DL-Met and L-Met are routinely used as supplements to meet requirements [31, 32]. In the present study, growth depression occurred when ducks were fed Met-deficient diets (Table 2), consistent with studies in broilers [33, 34]. Conversely, growth performance was improved, especially greater ADG and lower FCR, when diets were supplemented with crystalline DL-Met. As dietary Met increased, the FCR decreased linearly or quadratically, and ultimately the response reached a plateau. The Met deficient symptom of Pekin ducks was alleviated after feeding Met-supplemented diet, the explanation of which might be that the amino acids balance was ameliorated as increased dietary Met levels. Because of amino acids balance in diets, the utilization of other amino acids might be influenced by Met deficiency. For instance, dietary Met-to-lysine ratio affected Met metabolism in pregnant sow [35]. Dietary Met requirements of Pekin ducks from 15 to 42 days of age for optimal FCR were 0.38% and 0.42%, respectively, based on the linear broken line and quadratic broken line regression models (Table S2), close to the Met requirement (0.40%) recommended by Meat Duck Feeding Standard of China (NY/T 2122-2012) [36], but greater than the recommendation (0.30%) by NRC (1994) [24]. Therefore, we inferred that the latter recommendation was underestimated.

Carcass traits have been considered crucial parameters to evaluate dietary amino acid status [11, 32, 37], representing partly protein and fat deposition in ducks. Dietary Met level affected carcass composition in poultry and Met deficiency reduced edible carcass components and increased non-edible offal [11]. In our study, the eviscerated carcass yield of ducks at 42 days of age was increased, whereas subcutaneous and abdominal fat yields (weight and percentage) were decreased with increasing dietary Met levels, consistent with our previous studies [11, 32]. Dietary Met deficiency significantly changed lipid metabolism-related serum biochemical parameters (Fig. 2). That dietary Met deficiency caused an excessive accumulation of abdominal or subcutaneous fat was reported in broilers [38, 39], ducks [32] and goslings [40] and attributed to the explanation of which perhaps was increased adipogenesis and decreased lipolysis by dietary Met deficiency in poultry [41]. Clearly, lipid metabolism in Pekin ducks is affected by dietary Met status. Indices of fat deposition, e.g. abdominal and subcutaneous fat, were considered response indicators to evaluate Met requirements of ducks, with dietary Met requirements of 0.43% and 0.44%, respectively (Table S2). Based on growth performance, fat deposition and estimated Met requirements of Pekin ducks, Met-deficient and Met-adequate were selected to characterize for further studies on molecular mechanisms of lipid metabolism and fat deposition.

Dietary Met has critical roles in protein and lipid metabolism in ducks. In the present study, the relationship between dietary Met level and lipid metabolism, and mechanisms of fat deposition caused by dietary Met deficiency were explored. Dietary Met deficiency increased of yield of abdominal and subcutaneous fat (Table 3), accompanied by with increased serum TG and NEFA concentrations and decreased TCHO and low-density lipoprotein cholesterol (LDLC) concentrations (Fig. 1), clear evidence that which indicated dietary Met status altered the lipid metabolism in Pekin ducks. Subsequently, based on hepatic proteomic analysis, metabolic pathways of glycolysis/gluconeogenesis, fatty acid metabolism, TCA cycle, and pyruvate metabolism were significantly enriched (Fig. 3d). Furthermore, the PPAR signaling pathway had large differentially expressed proteins associated with lipid metabolism that were enriched in fatty acid transport and fatty acid oxidation (Fig. 3d, Table 4). Both proteomic and Western blot analyses both indicated ALB expression was significantly diminished in the Met-D group (Table 4, Fig. 4); this was verified by 2 in vitro hepatocyte models (Fig. 6). Similarly, serum ALB concentration was decreased in ducks fed a Met-D diet (Fig. 1g), consistent with dietary Met supplementation in broiler tending to increase serum albumin concentrations [42]. The serum ALB secreted by the liver and has an essential role in fatty acid transportation in blood [43]. As HSL and ATGL hydrolyze TG in adipocytes, NEFAs are released from the adipocyte into the blood, where they noncovalently combine with serum ALB. Insoluble NEFAs bound to soluble protein are carried to target tissues, e.g., liver, skeletal muscle, and cardiac muscle. In target tissues, dissociated NEFAs from albumin pass through cell membranes into cells to serve as fuel [43]. Currently, the exact mechanism of how fatty acids are moved into cells is not clear, although many studies have demonstrated the vital roles of fatty acid transporters, e.g., FATPs, FABPs and ACBP, in the entry of fatty acids into cells [44–46]. Stahl et al. [47] hypothesized a model for cellular fatty acid uptake. Long chain fatty acids could be transported across the cell membrane by FATPs and CD36, then fatty acids were rapidly esterified into acyl-CoA by ACSL to avoid outflow, with fatty acids and acyl-CoA rapidly bound by FABPs and ACBPs, respectively. Intracellular FABPs bind with fatty acids to form a fatty acid pool to prevent efflux of free fatty acids and to stabilize free fatty acids at low concentrations. In our study, hepatic FATP5, ACBP and ACSL5 expression of ducks fed Met-D diet were significantly diminished, whereas L-FABP expression was significantly enhanced (Fig. 4, Table 4), indicating that processes of fatty acids passing through cell membrane and intracellular fatty acids and acyl-CoA transportation were depressed. The enhanced L-FABP expression was probably explained by excessive accumulation of intracellular free fatty acids that increased L-FABP expression. The verification of Western blot of L-FABP expression agreed with proteomics results, whereas the *L-FABP* mRNA expression by qRT-PCR was discrepant (no difference between Met-D and Met-A groups), which suggested that dietary Met regulated hepatic L-FABP expression at the translational level. By analogy, in previous studies, FABP3 (H-FABP) expression was translationally rather than transcriptionally regulated in skeletal muscle of finishing pigs [48, 49]. As L-FABP and H-FABP are in same family proteins, we speculated that L-FABP was probably also regulated at a translational rather than transcriptional level.

Fat in animals is mainly derived from intestinal absorption and fat synthesis. However, in poultry, fat is synthesized mainly in liver [50] and transported to adipose or muscle tissues via very low-density lipoprotein (VLDL). During this process, hepatic proteins involved in fat synthesis (FASN, ACCα, SCD1) and fat transportation (Apo B, Apo AI) have vital roles [43]. However, based on proteomic analysis, hepatic expression of these proteins was not different between Met-D and Met-A groups (Table S3), suggesting Met deficiency may not influence hepatic fat synthesis and extrahepatic transportation in Pekin ducks. Additionally, total lipids, TG, TCHO and fatty acid concentrations in liver were not changed after feeding Met-D diet (Fig. S1). The fatty acids released from chylomicron and VLDL into adipocytes or other tissue cells, were re-esterified to TG for storage [43]. In the present study, the *LPL* mRNA expression in abdominal fat was significantly decreased in Pekin ducks fed a Met-D diet (Fig. 5), accounting for increased serum TG concentrations. The ATGL and HSL are two rate-limiting enzymes in TG lipolysis within adipocytes. Dietary Met deficiency decreased of ATGL and HSL expression (Fig. 5), which demonstrated TG lipolysis was depressed in abdominal fat in Pekin ducks.

*PPARα* could be largely expressed in liver; high expression contributes to decreased fat deposition and increased fatty acid oxidation and glycogen synthesis [51]. In fibroblasts, over-expression of L-FABP contributed to intracellular fatty acid transportation from the cytosol to the nucleus [52]; therefore, it has been speculated that L-FABP must enter the nucleus to promote fatty acid uptake [53]. In addition, L-FABP directly interacted with *PPARα* in the nucleus of primary hepatocytes [54], which might be due to fatty acids acting as ligands to activate *PPARα* [53]. In our study, hepatic *PPARα* mRNA expression was diminished (Fig. S2), whereas L-FABP expression was increased in the Met-D group (Fig. 4). Therefore, we inferred that decreased entry of fatty acids into the nucleus up-regulated expression of L-FABP in cytoplasm and down-regulated *PPARα* mRNA activated by fatty acids in the liver of Met-D ducks.

**Fig. 7:**
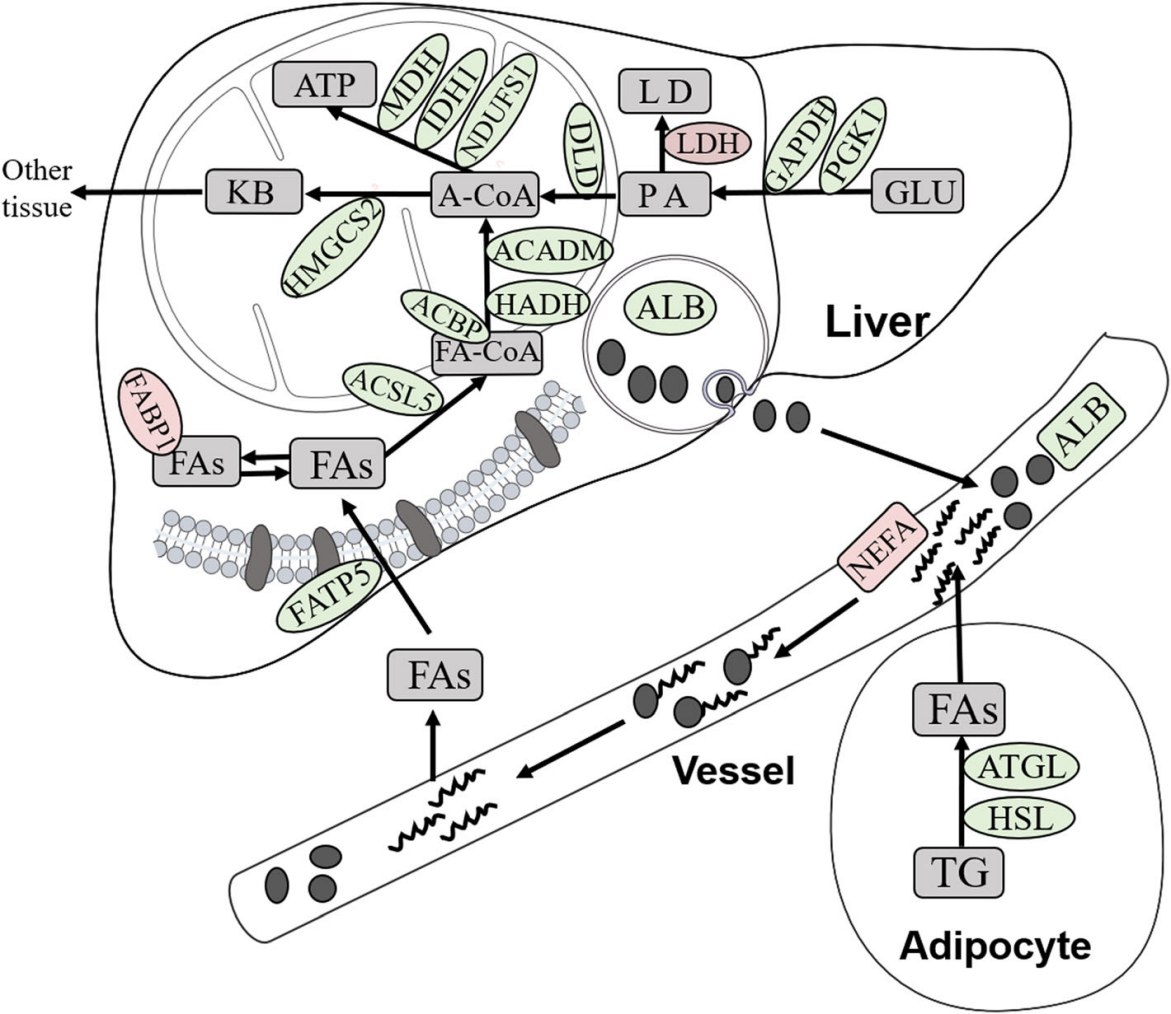
Summary of the differentially expressed proteins involved in lipid metabolism induced by dietary Met deficiency. Proteins involved in lipid metabolism that were affected by dietary Met deficiency; those in red were up-expressed, whereas those in green were down-expressed. FAs, Fatty acids; TG, Triglyceride; ALB, Albumin; FA-CoA, Acyl-CoA; A-CoA, Acetyl-CoA; KB, Ketone body; PA, Pyruvic acid; LD, Lactic acid; GLU, glucose; NEFA, Non-esterified fatty acid; LDH, Lactic dehydrogenase; L-FABP (FABP1), Fatty acid-binding protein, liver; ACBP, Acyl-CoA-binding protein; ACSL5, Acyl coenzyme A long-chain 5 synthetase; FATP5, Fatty acid transport protein 5; ATGL, Adipose triacylglyceride lipase; ACADM, Acyl-CoA dehydrogenase medium chain; NDUFS1, NADH:ubiquinone oxidoreductase core subunit S1; HSL, Hormone-sensitive lipase; ATP, Adenosine Triphosphate; MDH, Malate dehydrogenase; IDH1, Isocitrate dehydrogenase; DLD, Dihydrolipoyl dehydrogenase; HADH, Hydroxyacyl-CoA dehydrogenase; GAPDH, Glyceraldehyde-3-phosphate dehydrogenase; PGK1, Phosphoglycerate kinase; HMGCS2, 3-hydroxy-3-methylglutaryl coenzyme A synthase

Hepatic proteomics analysis revealed that key proteins involved in fatty acid oxidation, electron transport chain, TCA cycle, glycolysis/gluconeogenesis, and ketoplasia processes were impaired in Met-D ducks (Table 4), which probably resulted in insufficient ATP production, accompanying the greater LDH expression in liver. Conversely, decreased expression of HSL and ATGL proteins in abdominal fat implied adipocyte lipolysis was impaired, and decreased serum ALB concentrations led to deceleration of fatty acid transportation in response to Met deficiency. The above-mentioned explanation is a plausible, evidence-based explanation of mechanisms underlying growth retardation and excess fat deposition of Pekin ducks fed Met-D diets (Fig. 7).

## Conclusion

In the present study, dietary Met deficiency resulted in growth depression and excess fat deposition in Pekin ducks. Dietary Met deficiency affected gene and protein expressions associated with lipid metabolism and fatty acid transportation. The down-regulated genes and proteins related to lipid metabolism caused insufficient ATP production and stunted growth of Pekin ducks. Additionally, decreased expressions of ALB in liver and lipolytic genes and proteins in abdominal fat may have impaired of fatty acid transportation and further caused excessive fat deposition. We concluded that dietary Met deficiency stunts growth and increases fat deposition that might be related to suppression of fatty acid transportation and hepatic catabolism in Pekin ducks. Therefore, the present study provided new evidence regarding effects of dietary Met status on regulation of lipid metabolism in Pekin ducks.

## Supplementary Information

**Additional file 1: Table S1.** Sequences of primers for RT-qPCR. **Table S2.** Methionine requirements of Pekin ducks from 15 to 42 days of age based on linear broken-line models.

**Additional file 2: Table S3.** Differentially expressed proteins in livers of Pekin ducks at 42 days of age induced by dietary methionine deficiency. **Table S4.** The KEGG signal pathway of differentially expressed proteins

**Additional file 3: Fig. S1.** Effects of dietary methionine deficiency on hepatic lipid deposition in Pekin ducks at 42 days of age. **Fig. S2.** Effects of dietary methionine deficiency on PPAR genes expression in liver and abdominal fat of Pekin ducks at 42 days of age.

## Data Availability

All data generated or analyzed during the present study are available from the corresponding author on reasonable request. The datasets supporting the conclusions of this article are included in the main manuscript and supplemental materials.

## Abbreviations

Met: Methionine
Met-D: Methionine-deficient
Met-A: Methionine-adequate
ADG: Average daily gain
ADFI: Average daily feed intake
FCR: Feed conversion ratio (feed: gain)
TCHO: Total cholesterol
TG: Triglyceride
HDLC: High-density lipoprotein cholesterol
LDLC: Low-density lipoprotein cholesterol
NEFA: Non-esterified fatty acid
TP: Total protein
ALB: Albumin
ALT: Alanine aminotransferase
AST: Aspartate amino transferase
TBIL: Total bilirubin
DBIL: Direct bilirubin
ALP: Alkaline phosphatase
LDH: Lactic dehydrogenase
UA: Uric acid
GLU: Glucose
PPARα: Peroxisome proliferators-activated receptor alpha
PPARγ: Peroxisome proliferators-activated receptor gamma
L-FABP (FABP1): Fatty acid-binding protein, liver
A-FABP (FABP4): Fatty acid-binding protein, adipose
ACBP: Acyl-CoA-binding protein
ACSL5: Acyl coenzyme A long-chain 5 synthetase
FATP5: Fatty acid transport protein 5
LDHA: Lactate dehydrogenase A chain
FASN: Fatty acid synthase
LPL: Lipoprotein lipase
ATGL: Adipose triacylglyceride lipase
ACADM: Acyl-CoA dehydrogenase medium chain
NDUFS1: NADH:ubiquinone oxidoreductase core subunit S1
FAT/CD36: Fatty acid translocase
HSL: Hormone-sensitive lipase
p-HSL-S563: Phosphor-hormone-sensitive lipase at Ser563
TCA: Tricarboxylic acid cycle
VLDL: Very low-density lipoprotein
