# Supplementary material for: Dietary methionine deficiency stunts growth and increases fat deposition via suppression of fatty acids transportation and hepatic catabolism in Pekin ducks
Source: J Anim Sci Biotechnol. 2022 May 18;13:61. doi: 10.1186/s40104-022-00709-z (PMC9115956; doi:10.1186/s40104-022-00709-z)
Supplement: Supplementary file 1 — Additional file 1: Table S1. Sequences of primers for RT-qPCR. Table S2. Methionine requirements of Pekin ducks from 15 to 42 days of age based on linear broken-line models. [file 40104_2022_709_MOESM1_ESM.docx]

Table S1 Sequences of primers for real-time PCR

| Gene | Accession number | Primer sequences (5'-3') | Product length, bp |
| --- | --- | --- | --- |
| *β-actin* | NM_001310421.1 | F: GGTATCGGCAGCAGTCTTA | 158 |
|  |  | R: TTCACAGAGGCGAGTAACTT |  |
| *PPARα* | NM_001310383.1 | F: ACCAGCATCCAGTCCTTCATCCA | 146 |
|  |  | R: AACCTTCACAAGCATGTACTCCGTAA |  |
| *PPARγ* | NM_001310398.1 | F: CAGGTGTGATCTTAATTGTCGCATCC | 136 |
|  |  | R: CTCCTTCTCCGCTTGTGGCATTC |  |
| *L-FABP* | HQ640427.1 | F: GCACGGTGAACCTAGTAAATGGGAAG | 117 |
|  |  | R: CGAGCGTTACTCCACCAACAGTTAT |  |
| *A-FABP* | NM_001310375.1 | F: TTGATGAGACCACAGCAGATGACAG | 128 |
|  |  | R: GTTCCCATCCACCACTTTCCTCTTTA |  |
| *ACBP* | XM_027461098.1 | F: GAGGAGGTGAAGCAGCTCAAGTC | 111 |
|  |  | R: CATACCAGGGCGATCCGTGTTG |  |
| *ACSL5* | XM_005014458.4 | F: TGGTATGACTCTGAAGACGGCTGAA | 256 |
|  |  | R: TGTTGTCCAGTCTCCAGGCATTGA |  |
| *FATP5* | ENSAPLT00000001958.2 | F: CACCTGTGTCCTGCGTTCCAA | 144 |
|  |  | R: CCGTGCTTGTGGTCGTTGTCA |  |
| *ALB* | NM_001310394.1 | F: GCAAGGAATACGAGGACAACAGAGT | 114 |
|  |  | R: CATAATCAGCAGCCACACCAAGGA |  |
| *LDHA* | XM_027459308.1 | F: TGGGTGGATTGTTGGAGAGC | 165 |
|  |  | R: ACCTCATAGGCACTGTCCAC |  |
| *FASN* | NM_001310798.1 | F: GCTGAGAAACGCCAATACC | 181 |
|  |  | R: GAGCAAGACACCGCAAACT |  |
| *LPL* | FJ859348.1 | F: GACCAAGACCAACCAGCCATTC | 245 |
|  |  | R: CGCCTGACTTCACTCTGACTCT |  |
| *ATGL* | NM_001310387.1 | F: CCCTGTGTACTGCGGGCTGATA | 220 |
|  |  | R: GCGGTAGAGGTTGCGAAGGTTG |  |
| *ACADM* | XM_038171570.1 | F: AGACCAGACCACCTGTAGCA | 298 |
|  |  | R: CCTCCAAAAATCTGCACCGC |  |
| *CD36* | XM_005016712.4 | F: CGTTCGCATCACCAGTTGAAGTTG | 104 |
|  |  | R: GCAGGCACTAATATCTAGGACTCCAG |  |

*PPARα*, oxidosome proliferator activated receptor; *PPARγ*, oxidosome proliferator activated receptorγ; *L-FABP*, fatty acid-binding protein, liver; *A-FABP*, fatty acid-binding protein, adipose; *ACBP*, acyl-CoA-binding protein; *ACSL5*, acyl coenzyme A long-chain 5 synthetase; *FATP5*, fatty acid transport protein 5 (also known as SLC27A5, solute carrier family 27 member 5); *ALB*, albumin; *LDHA*, lactate dehydrogenase A chain; *FASN*, fatty acid synthase; *LPL*, lipoprotein lipase; *ATGL*, adipose triacylglyceride lipase; *ACADM*, acyl-CoA dehydrogenase medium chain; *FAT/CD36*, fatty acid translocase; *LDHA*, lactic dehydrogenase A.

Table S2 Methionine requirements of Pekin ducks from 15 to 42 days of age by linear broken-line models

| Assessment index | Models | Regression equation^1^ | Estimated L^2^ | Met requirement^2^ | 95% Confidence Interval | R^2^ | *P* value |
| --- | --- | --- | --- | --- | --- | --- | --- |
| FCR | Linear broken-line | *y*=1.93+2.27×(0.38-*x*) | 1.930±0.006 | 0.38±0.006 | 0.347-0.402 | 0.995 | 0.0055 |
|  | Quadratic broken-line | *y*=1.93+10.7×(0.42-*x*)^2^ | 1.930±0.006 | 0.42±0.015 | 0.358-0.483 | 0.995 | 0.0055 |
| Abdominal fat weight | Linear broken-line | *y*=20.1-79.3×(0.43-*x*) | 20.1±0.14 | 0.43±0.003 | 0.418-0.444 | 0.999 | 0.0007 |
| Abdominal fat percent | Linear broken-line | *y*=0.87+4.0×(0.43-*x*) | 0.87±0.02 | 0.43±0.008 | 0.394-0.460 | 0.995 | 0.0048 |
| Skin and subcutaneous fat percent | Linear broken-line | *y*=21.6+21.3×(0.44-*x*) | 21.6±0.04 | 0.44±0.003 | 0.424-0.452 | 0.999 | 0.0007 |

^1^Linear broken-line equation is *y* = l+u∗(r-*x*); quadratic broken-line equation is *y* = l+u∗(r-*x*)^2^, when *x* ≤ r, and *y* = l when *x* > *r*; *x* = independent variable, *r* = Met requirement, *y* = dependent variable, l = theoretical maximum (minimum), u = rate constant.

^2^Expressed as Means ± SE.
