# Supplementary material for: Dietary methionine deficiency stunts growth and increases fat deposition via suppression of fatty acids transportation and hepatic catabolism in Pekin ducks
Source: J Anim Sci Biotechnol. 2022 May 18;13:61. doi: 10.1186/s40104-022-00709-z (PMC9115956; doi:10.1186/s40104-022-00709-z)
Supplement: Supplementary file 3 — Additional file 3: Fig. S1. Effects of dietary methionine deficiency on hepatic lipid deposition in Pekin ducks at 42 days of age. Fig. S2. Effects of dietary methionine deficiency on PPAR genes expression in liver and abdominal fat of Pekin ducks at 42 days of age. [file 40104_2022_709_MOESM3_ESM.docx]

**
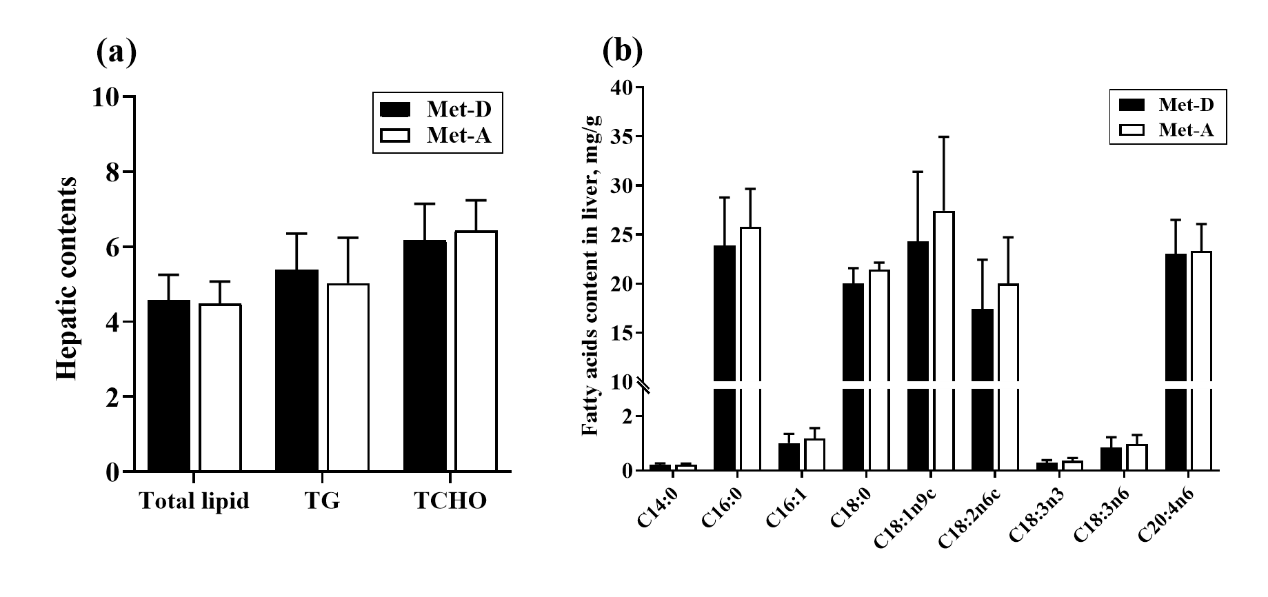
Fig. S1.** Effects of dietary methionine deficiency on hepatic lipid deposition in Pekin ducks at 42 days of age. (a) The total lipid (%), triglyceride (TG, mmol/g liver), total cholesterol (TCHO, mmol/g liver) and (b) fatty acids (mg/g) contents. Results are presented as means with plus error bars (standard deviation). Differences were assessed by Student t test (*n* = 6).


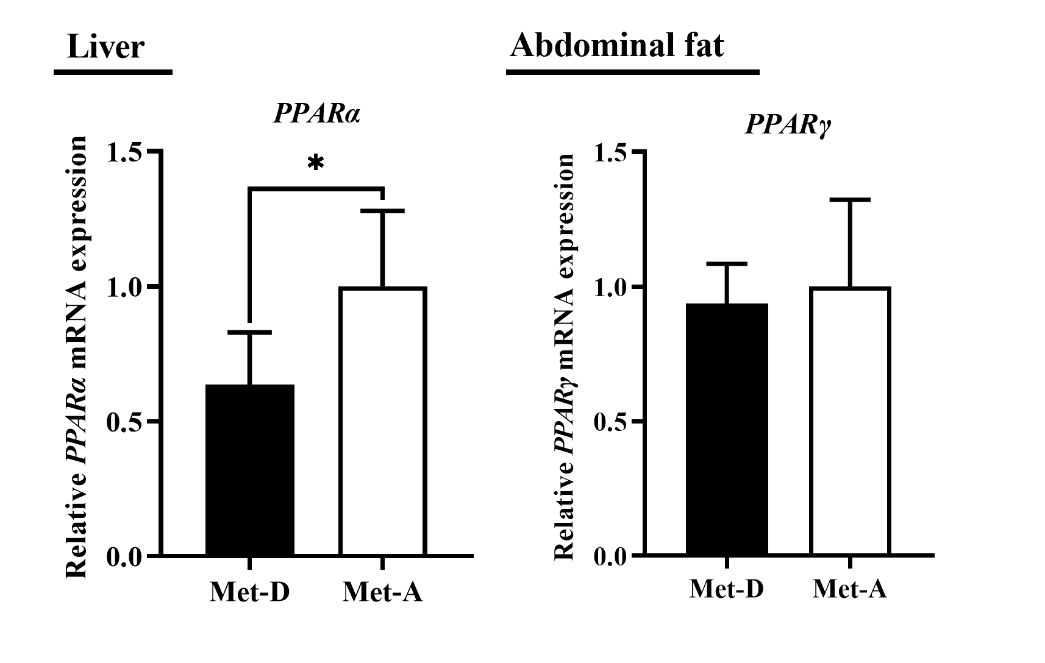


**Fig. S2.** Effects of dietary methionine deficiency on mRNA of *PPARα* in liver and *PPARγ* in abdominal fat of Pekin ducks at 42 days of age. Results are presented as means with plus error bars (standard deviation). Results are presented as means with plus error bars (standard deviation). Differences were assessed by Student t test (*n* = 6) and denoted as follows: * *P* < 0.05.
